# Biological Risks and Laboratory-Acquired Infections: A Reality That Cannot be Ignored in Health Biotechnology

**DOI:** 10.3389/fbioe.2015.00056

**Published:** 2015-04-28

**Authors:** Ana Cláudia Coelho, Juan García Díez

**Affiliations:** ^1^Department of Veterinary Sciences, Veterinary and Animal Science Center (CECAV), School of Agrarian and Veterinary Sciences, University of Trás-os-Montes e Alto Douro, Vila Real, Portugal

**Keywords:** biological risks, biosafety, biotechnology, laboratory-acquired infections, health

## Abstract

Advances and research in biotechnology have applications over a wide range of areas, such as microbiology, medicine, the food industry, agriculture, genetically modified organisms, and nanotechnology, among others. However, research with pathogenic agents, such as virus, parasites, fungi, rickettsia, bacterial microorganisms, or genetic modified organisms, has generated concern because of their potential biological risk – not only for people, but also for the environment due to their unpredictable behavior. In addition, concern for biosafety is associated with the emergence of new diseases or re-emergence of diseases that were already under control. Biotechnology laboratories require biosafety measures designed to protect their staff, the population, and the environment, which may be exposed to hazardous organisms and materials. Laboratory staff training and education is essential, not only to acquire a good understanding about the direct handling of hazardous biological agents but also knowledge of the epidemiology, pathogenicity, and human susceptibility to the biological materials used in research. Biological risk can be reduced and controlled by the correct application of internationally recognized procedures such as proper microbiological techniques, proper containment apparatus, adequate facilities, protective barriers, and special training and education of laboratory workers. To avoid occupational infections, knowledge about standardized microbiological procedures and techniques and the use of containment devices, facilities, and protective barriers is necessary. Training and education about the epidemiology, pathogenicity, and biohazards of the microorganisms involved may prevent or decrease the risk. In this way, the scientific community may benefit from the lessons learned in the past to anticipate future problems.

## Introduction

Health biotechnology and bioengineering have recently undergone major advances in both human and animal medicine. They involve the study and manipulation of modified living organisms, genetically modified organisms (GMOs), transgenic plants, animals or microorganisms, and the production of vaccines carried out in special laboratories with different biosafety and biosecurity levels according to the pathogenicity of the organisms under study (Mattiasson, 2013) Since microorganisms and GMOs may be harmful or pathogenic to animals and humans, the possibility of enhanced virulence through genetic manipulation or increased drug-resistance, among other routes, implies putting specific biosafety and biosecurity procedures in place. To guarantee public health, every possible scenario of an outbreak arising through the release of a bio-hazard into the environment cannot be neglected (Nordmann, 2010).

Biosecurity includes a set of preventive measures that vary according to the organisms which are under study. Biosafety and biosecurity are terms which are frequently referred to with similar meanings in the literature. While the differences between the two concepts have been specified academically, in practice, when one is actually working hands-on in a laboratory it is more difficult to draw such distinctions as referred by Daoust-Maleval (2011). Moreover, Bakanidze et al. (2010) indicates that concepts of biosafety and biosecurity include several control measures that may overlap each other. To avoid misunderstanding, biosafety includes all the prevention measures carried out to avoid the infection with pathogenic organisms and/or toxins and their release to the environment (OMS). In the other hand, biosecurity is referred to all the preventive measures to avoid or reduce the risk of transmission of infectious diseases in crops and livestock, quarantined pests, or GMOs (Baltz et al., 2010). The objective of this review is to highlight the main risks associated with biological investigation in laboratory, the potential laboratory-acquired infections (LAIs) by laboratory personnel, and suggested recommendations to avoid them.

## Biotechnology in Health and Biosafety

Advances and research in biotechnology have applications over a wide range of areas, such as microbiology, medicine, the food industry, waste management, agriculture, GMOs, and nanotechnology, among others (Thompson, 2007; Baltz et al., 2010; Bennett et al., 2013).

Biosafety cuts across different human and veterinary science. The first infectious diseases acquired in a laboratory were reported at the time of Pasteur and Koch in 1890. However, several decades passed before the connection between human diseases and the handling of pathogenic microorganisms was understood (Sulkin, 1961; Traxler et al., 2013), and the implementation of protective measures against biological risks in humans was reported in the literature (Sulkin, 1961; Pike, 1978; Collins and Kennedy, 1998). The first safety measures in microbiology laboratories that work with pathogenic microorganisms were implemented in North America and the United Kingdom at the beginning of the 1970s. These measures included laboratory training and education about the correct use of personal protective equipment and physical containment measures designed to limit the potential spread of biological agents (Frommer et al., 1989). After that, safety measures were applied in laboratories that work with GMOs. One of the most important considerations in the development and application of biotechnology is the maintenance of both biosafety and biosecurity measures at high levels. The Cartagena Protocol on Biosafety is an international regulation of the use of GMOs resulting from modern biotechnology. This agreement, which focuses specifically on the transboundary movement of GMOs, promotes biosecurity by establishing rules and procedures for the safe transfer, handling, and use of GMOs (McKenzie and Ascencio, 2003). Since biotechnology could be a potential threat to the population, the Biological Weapons Convention – a multilateral disarmament treaty focused on the prohibition of biological and toxins weapons (Millett, 2006; Bakanidze et al., 2010) – recognized the application of biosafety and biosecurity measures as tools to prevent the development, acquisition, or use of biological and/or toxins weapons. Biotechnology investigations have developed a wide range of powerful tools and processes to improve human and animal health. Good laboratory practices regarding biosafety and bioethics are essential measures to avoiding LAIs. Some publications have evaluated the effectiveness of biosafety and biosecurity measures in traditional laboratories (Kimman et al., 2008; Nasim et al., 2010). Monitoring laboratory infections is one of the most important methods of evaluating the effectiveness of containment measures (McLean et al., 2002).

## Biological Risk Classification

Pathogenic microorganisms represent only a small proportion of the biological hazards of concern in laboratory investigations. Their control importance is based on their potential threat to humans, animal populations, and also agriculture. In addition, they can be responsible for large-scale infections with huge economic costs and environmental consequences (Bosia, 2008).

In addition, new viruses are constantly emerging that threaten the lives of humans and animals. All laboratory staff who work with biological samples are exposed to a number of infectious materials and subject to risk from clinical specimens and cultures. Since hazardous agents can be transmitted in the laboratory by inhalation, inoculation, or through the skin, among others, it is necessary to know the characteristics of the agents in study according to their risk to the health of laboratory staff, and to the human and animal population in case of an outbreak (Corrao et al., 2012).

The World Health Organization (WHO) developed a system to classify microorganisms based on their danger to laboratory staff and the public (World Health Organization (WHO), 2004). Biological agents of risk group 1 include those unlikely to cause disease in man, biological agents of risk group 2 include those that can cause disease in humans and pose a danger for workers with little chance of spreading among them or to the community. In addition, there is prophylaxis and effective treatment available to the community. Biological agents of risk group 3 include those that can cause serious illness in humans, represent a serious danger to workers with risk of spreading to the community, and there is an effective treatment or prophylaxis. Finally, the biological agents of risk group 4 include those that can cause severe disease in humans and represent a serious danger to workers, with likelihood of being spread to the community and there is usually no effective prophylaxis or treatment (World Health Organization (WHO), 2004).

However, there are other biological risk classifications by country, based mainly on national policies. In the European Union, Directive 2000/54/EC (2000) on the protection of workers from risks through exposure to biological agents, the agents are classified into four risk groups based on the risk level of infection. In the United States, biological hazards are also classified into four risk groups, from minimal hazard (risk group 1) to those responsible for very serious diseases (risk group 4), as described by the Centers for Disease Control and Prevention (CDC) (Center for Disease Control and Prevention (CDC), 2009). In other countries such as Canada or Australia, pathogenic agents are also classified into four risk groups (NZS, 2002; PHAC, 2013).

Taking into account the pathogenic effects to humans and the potential danger to the environment, the classification of pathogenic agents as well as their containment measures should be undertaken with a single goal: to reduce all potential risks (Kimman et al., 2008). Moreover, investigation with infected transgenic animals that carry the genes of an infectious agent must be subjected to a proper risk evaluation and also subject to correct handling with specialized containment facilities and equipment. The specialized containment can address the risks of aerosolized particles, such as in studies of tuberculosis or lymphocytic choriomeningitis, and animals such as poultry infected with influenza (World Health Organization (WHO), 2004; OIE, 2012; Shinnick and Gilpin, 2012).

## Biological Risks to Health in Biotechnology Laboratories

There are many biological risks in health biotechnology, such as bacteria, viruses, rickettsiae, fungi, and parasites (Liberman et al., 1990; World Health Organization (WHO), 2004). Regarding biosafety and biosecurity in biomedical laboratories, there is great concern about new vaccines, diagnostic tools, or therapeutic agents, some of which are made by genetic engineering (Doblhoff-Dier and Collins, 2001). Currently, a main concern of biosafety is due to the emergence of new diseases or the re-emergence of diseases that were already under control (Brown, 2004; Jones et al., 2008). In laboratories, there are many tasks that involve numerous risks to the laboratory staff. Thus, any incident associated with a given microbiological hazard is probably most likely to happen in a microbiology laboratory. However, incidents are not associated to a single factor but the interaction of several of them (Sewell, 1995; Kozajda and Szadkowska-Stanczyk, 2010).

In the near future, advances in microbiology associated with biotechnology will increase the knowledge of viroids, viruses, and bacteria cells that carry novel genetic material, which has been modified or constructed through genetic engineering. Thus, new concerns in biosecurity and environmental health will emerge. Currently, there is an urgent demand for new vaccines against extremely hazardous pathogens such as Ebola (Levine et al., 2014). The research will involve the manipulation of pathogenic microorganisms that could have harmful effects on public health and the environment. To guarantee the biosafety of laboratory staff and the biosecurity measures of the facilities, the intrinsic and potentially harmful characteristics of all microorganisms under study must be identified.

Although research in biotechnology is necessary, nowadays there is a dilemma about the freedom or limitation of these investigations. Thus, gain-of-function (GOF) research or dual use research (DUR) have arisen as an important concern, not only among the scientific community but also among the population (Casadevall and Imperiale, 2014). While GOF is associated with the acquirement of a new activity or the enhancement, a previous function, DUR, is associated with a misuse of science (Suk et al., 2011; Duprex et al., 2015). Research with highly pathogenic microorganisms, like H5N1 influenza, anthrax among others, could derive into a serious biological threat to a population or even terrorism (Resnik, 2010; Lipkin, 2012). Epidemics of pandemic proportions or improved previous research to develop bio weapons could be an uncontrollable risk for a population. As a result, both GOF research and DUR must be regulated with strong biosafety measures, restricted research, and specific policy as was recently discussed in Germany (Karberg, 2014).

### Vectors used in gene therapy

Gene therapy, *in vivo* and *ex vivo*, use vectors classified as viral or non-viral, which express the gene or genes of therapeutic interest. Non-viral vectors include liposomes, naked DNA, and DNA-protein complexes (Doblhoff-Dier and Collins, 2001), while viral vectors derived from viruses that are attenuated in order to prevent destructive infection in target tissues. Non-viral vectors are preferred from the biosafety point-of-view, although they are less efficient than viral. Thus, viral vectors are usually used to avoid this disadvantage. The expression of the information encoded in genes leads to functional modification of infected cells, and the evaluation of these modifications are extremely complex and incomplete when the modified cells belong to the host. The safety of gene therapy vectors has been the focus of regulatory attention by committees. They are required to assess the proportionality between the magnitude of the risks and potential therapeutic benefits, as well as to monitor the occurrence of risks in the experiment once they have been approved (Verma and Somia, 1997). Biosafety in laboratories with gene therapy manufacture must consider the staff and the patients that may be in contact. In addition, a comprehensive risk assessment of viral vectors must be carried out.

Ideally, viral vectors should be designed only to act as transporters of the exogenous genetic material to avoid the development of new replicative viral particles in transduced cells. Thus, it is understood that a viral vector cannot make any escape or reversion in virulent forms. The biohazard of these vectors is determined by their own biological risk to the laboratory staff, by the environment of the exogenous genes they carry and also by the tropism of the recombinant virus (Doblhoff-Dier and Collins, 2001).

Gene therapy, due to its technical and ethical characteristics, is regulated by law. In the United States, the National Institutes of Health (1999) and the Food and Drug Administration (1998) published several guides to describe the main biosecurity measures with which all laboratories must comply.

Utilization of retroviral vectors must be subjected to a previous risk assessment. Since they are highly infectious and may infect and propagate themselves in human cells, they may be considered as an important biohazard to the laboratory staff (Thomas et al., 2003; Anson, 2004).

The principal biosafety concern when working with retroviruses is the chance of viruses entering the cells and tissues of laboratory personnel through skin lesions or by accident. The closer the contact the greater the risk, because retroviral particles are extremely labile and short-lived. Even when using defective retroviruses that present a lower risk of infection, laboratory staff must be trained in virology and tissue culture (Le Duc et al., 2008). Handling of animals infected with retroviruses, especially those that present a potential human cell risk, need high standards of biosecurity measures, particularly for avoiding potential animal escape and the spread of infection. Additionally, when handling infected animals extreme caution must be taken to avoid injuries through bites, clawing, or scratches. Laboratory staff should only be allowed to handle infected animals if they have specific training and always under the supervision of a senior (Wolfensohn and Lloyd, 2013).

Regarding gene therapy, immunomodulation has arisen as a new trend in biotechnology. It can be defined as therapeutic procedures aimed at modifying the immune response (Gea-Banacloche, 2006). Research in immunomodulation is focused on correcting specific diseases or immuno-deficiencies such as HIV, cancer, allergies, or inflammatory diseases among others. It consists in the hybridization of synthetic genetic sequence in the organisms targeted to express or suppress specific genes in the host. Several oligonucleotides based on DNA or RNA has been used, vaccination being the most effective immunomodulatory technique (Gea-Banacloche, 2006). However, the potential biological risk of immunomodulation is associated with the uncertain hybridization and gene expression in the host, which may cause an adverse effect (Vo et al., 2006). Thus, the main biosafety concern in the laboratory is associated with accidental needlesticks that can cause unpredictable responses in an organism. However, the utilization of oligonucleotides sequences of RNAi or siRNA increases the control of the gene expression (Behlke, 2006), and further research is necessary.

### Vaccines

The manufacture of vaccines based on viral or bacterial recombinants are the most common approaches to live GMO vaccines, and are sources of biological risk in biotechnology laboratories. Several pathogens, including bacteria like *Lactococcus lactis* or *Salmonella Typhymurium* have been used in the manufacture of recombinant vaccines (Robinson et al., 1997; Gomez-Duarte et al., 1998). Recombinant viral vaccines are manufactured from poxvirus, paramixovirus, adenovirus, or vaccinia virus, among others (Rotz et al., 2001). The large-scale manufacture of vaccines, as well as other biological products, should be considered as a potential occupational risk factor. Thus, the training and education of workers engaged in vaccine manufacture must be a priority (Doblhoff-Dier and Collins, 2001). There are some biosafety guidelines about manufacture of recombinant vaccines, laboratory procedures, exposure of laboratory staff, or advice about outbreak, among others (Doblhoff-Dier and Collins, 2001; World Health Organization (WHO), 2004). For the most part, viral vectors for vaccine development have similar biosafety risks as gene therapy viruses, as we previously described. The major differences in risk are associated with the antigens or immune enhancing components of the vaccine and challenge experiments (Vemula and Mittal, 2010).

### Xenotransplantation

Another possible biohazard in biotechnology is associated with xenotransplantation. This term includes any procedure that involves the transplantation, implantation, or infusion of human cells, tissues, or organs from non-human animals (Yang and Sykes, 2007). Nowadays, biotechnology companies develop genetically engineered pigs to meet the demand for organs, which are compatible with humans. Transgenic pigs have been adopted as organ donors rather than primates, because of ethical objections to the use of primates (Lambrigts et al., 1998). Moreover, almost all primate species are protected. Since xenotransplantation may imply a potential zoonotical risk, donor animals must not carry any potential zoonotical disease, with special care for immunosuppressed patients (Michaels et al., 1994). Potential virus infections associated with xenotransplantation have been widely discussed by the scientific community (Tacke et al., 2000; Fishman, 2014).

Furthermore, xenotransplantation has been criticized by the scientific community due to the risk of new viral infections with zoonotical characteristics that can infect the population (Boneva and Folks, 2004). Viral infection from pig to human cells, and also from xenotransplantation of baboon livers to humans, have been reported (Allan, 1998; Denner, 2008). The DNA of the simian foamy virus (SFV) and the baboon endogenous virus (BaEV) have been observed in many tissues of patients. The presence of baboon mitochondrial DNA (evidence of baboon cells) suggests that baboon leukocytes may carry latent or active viral infections (Allan, 1998). Other works suggest that pig endogenous retrovirus (PERV) can infect culture cells of human origin, and after completing its life-cycle in human cells it is able to infect other human cells (Patience et al., 1997). Because pigs present multiple copies of PERVs, breeding PERV-free pigs is almost impossible (Boneva and Folks, 2004; Denner and Tönjes, 2012).

### Transportation of infectious and biological material

Transportation and transfer of infectious and biological materials is an important biological risk and an occupational risk to staff through improper packaging (Snyder, 2002; Kozajda et al., 2013). There are several guidelines concerning the transport of infectious material (World Health Organization (WHO), 1997). Thus, shipping of biohazard material within and between facilities must be carried out with extreme care. GMOs must be transported in securely sealed triple package systems to avoid any escape. In addition, the primary and secondary package must be previously decontaminated (World Health Organization (WHO), 2012).

These units should ideally be placed within sturdy outer shipping containers. The removal of wastes and by-products from genetic engineering labs requires comparable packaging and container specifications. Furthermore, pathogenic or infectious microorganisms must be wholly contained inside a sealed, unbreakable primary container (World Health Organization (WHO), 2012). Training and education in packing and shipping hazardous laboratory material is necessary for all laboratory staff (Biosafety in Microbiological and Biomedical Laboratories (BMBL), 2009; World Health Organization (WHO), 2012).

### Biosafety in research with experimental animals

Today, research with experimental animals must be carried out according to specific policies to guarantee their protection (Biosafety in Microbiological and Biomedical Laboratories (BMBL), 2009; Directive 2010/63/EU, 2010). However, their utilization involving the use of hazardous biological agents also represents an important biosafety issue. According to the potential risk, facilities at an animal laboratory are classified into three categories as reported by the CDC (Biosafety in Microbiological and Biomedical Laboratories (BMBL), 2009), although several countries also present specific legislation. As previously described, it is necessary to identify the potential hazards associated with the animal species and the pathogens used (OIE, 2012). Moreover, it is important to differentiate the zoonotical agents from the non-zonootical pathogens. The first represent a potential threat to the laboratory staff, whereas non-zoonotical pathogens, transgenic animals, or GMOs inoculated in those animals are a threat to the environment (Peeters, 2011). Although laboratory research with experimental animals involves a large variety of pathogenic organisms, such as bacteria, virus, parasites, or prions, among others, reports on laboratory-acquired or zoonotical-acquired infection are scarce. To avoid potential LAIs and/or the escape of animals used in research, animal facilities must meet specific requirements. Operations with research animals must be carried out in individually ventilated cages that guarantee isolation between animals and laboratory staff (Peeters, 2011). To improve the biosafety of the laboratory staff, workers needs to operate with personal protective equipment within facilities with an appropriate biosecurity level. Moreover, in an animal facility, different types of wastes such as feces, bedding, manure, or carcasses must be appropriately removed and processed (Schultz, 2004).

## Laboratory-Acquired Infections

The term LAIs refer to all infections acquired through laboratory work or laboratory-related activities with or without the onset of infections, and result from occupational exposure to infectious agents (Pike, 1976; Wei et al., 2011). There are only a few reports of laboratory acquired infections and accidents with GMOs (Kimman et al., 2008). LAIs can occur in biological facilities such as microbiological or animal facilities during research and investigations. In addition, the higher the biosecurity level of the laboratory, the stronger the biosafety and biosecurity measures are. Although LAIs and outbreaks associated with risk group 4 are extremely rare, BSL-4 laboratories are necessary to investigate new emerging diseases or bioterrorism threats (Nisii et al., 2013). Identification of a laboratory outbreak is difficult, and therefore training and education is necessary to avoid outbreaks (Risi et al., 2010). The determination of the source of infection in a laboratory worker can be difficult, because the etiological agent is sometimes present in the laboratory and outside the workplace in the population as well.

In addition, LAIs are an important issue in regard to public health, because an infected worker could be a transmission risk for other people. However, the literature about LAIs is quite scarce (Pike, 1978; Traxler et al., 2013). LAIs still occur, although comprehensive reports on LAIs are few, and based on internal reports of the infection laboratory or by official investigation. The underreporting of LAIs is widely acknowledged, and is ascribed to the negative consequences for a company or authority to which the laboratory belongs (Sewell, 1995). On the other hand, several reports about LAIs in traditional laboratories have been published in the recent years (Britton et al., 2011; Riyesh et al., 2014).

The most common routes of infection are inhalation (particularly by aerosols), percutaneous inoculation (needlestick injuries, broken glass injury, and/or animal bites or scratches), direct contact between contaminated surfaces (gloves, hands), and mucous membranes as well as through ingestion – for example by smoking, eating, or accidental aspiration through a pipette (Kozajda et al., 2013; Traxler et al., 2013).

The risk assessment of the potential infection of laboratory staff must consider the route of transmission and also the minimal infective dose for humans, which varies according to the route of inoculation (Johnson, 2003). The increased risk for microbiology laboratory staff that do research with zoonotical agents has long been recognized. Although LAIs caused by pathogenic bacteria have been described as the most common, LAIs caused by viruses have arisen nowadays (Singh, 2011).

### *Brucella* spp.

Brucellosis has been reported as one of the most important LAIs (Traxler et al., 2013).

Most infections were acquired through workers being unaware of contaminated cultures from clinical cases (Bouza et al., 2005; Knudsen et al., 2013; Dentinger et al., 2014).

Brucellosis is still recognized as an important LAI (Knudsen et al., 2013), and outbreaks have been mainly associated with the improper use of the biological safety cabinets (BSC) and deficient recognition of *Brucella* spp. isolated by laboratory staff (Bouza et al., 2005; Dentinger et al., 2014). *Brucella* spp. belongs to risk group Level 3. In contrast, laboratory-acquired brucellosis is not always associated with an occupational accident but due to direct contact, contamination of skin, splashing in the mucous membranes or conjunctivae, or needlestick injuries (Kiel and Khan, 1993; Ergönül et al., 2004).

There have been reported cases of infections due to eating and drinking near a culture-processing bench, and the lack of individual protection equipment when handling infectious material (Ergönül et al., 2004; Singh, 2009). Due to scarce reports about laboratory-acquired brucellosis, it is difficult to quantify (Knudsen et al., 2013) and difficult to assess, because of a lack of surveillance and central reporting based mainly on case reports. Unawareness of the pathogenicity of *Brucella* spp. and deficient training in regard to handling biohazard materials may contribute to new outbreaks (Sam et al., 2012). Furthermore, the low infectious dose of *Brucella* spp. can cause laboratory outbreaks that affect the laboratory staff, ranging 30–100% (Yagupsky and Baron, 2005).

### *Mycobacterium* *tuberculosis*

Early surveys of laboratory acquired tuberculosis found three to nine times greater incidence of *Mycobacterium tuberculosis* among laboratory personnel than in the general population (Singh, 2009). However, laboratory-acquired tuberculosis is difficult to trace due to the environmental distribution of the pathogenic microorganisms as well as the chronicity of infection, because infection could happen outside of the workplace (Collins and Grange, 1999). The greatest risk of LAI for laboratory personnel who handle *M. tuberculosis* is associated with the generation of aerosols. Moreover, *M. tuberculosis* is also characterized by a low infective dose for humans (Center for Disease Control and Prevention (CDC), 2009). There are reported cases of *M. tuberculosis* associated with inadequate isolation procedures and high volumes of specimens handled (Kao et al., 1997). To avoid possible LAI by *M. tuberculosis*, mycobacteria must be handled in class II or III BSC (Weinstein and Singh, 2009).

### LAIs associated to other bacterial agents

Other bacterial agents have been associated with LAIs with lower frequency. Among them, *Francisella tularensis* is a zoonotical infection, usually presented as an ulceroglandular form but also as pneumonia. *F. tularensis* is characterized by its high infectivity via aerosols and the severity of disease in humans. LAIs by *F. tularensis* have been reported in the literature, and are more frequently associated with cultures than with clinical material or infected animals (Shapiro and Schwartz, 2002; Singh, 2009). Since antibiotic therapy treatment presents some adverse effects, a combination of vaccination and correct biosafety measures has been referred to as the most valuable control tool (Lam et al., 2012).

Enterobacteriaceae group microorganisms such as *Salmonella* or *Shigella* have been reported as LAIs (Mermel et al., 1997; Baron and Miller, 2008). The increasing number of antimicrobial agent-resistant pathogens may influence these infections. Thus, other pathogenic bacteria like *Clostridium difficile, Escherichia coli*, or *Klebsiella* spp. have been related with LAIs (Coia, 1998; Bouza et al., 2005).

### *Neisseria* *meningitidis*

Occupationally acquired meningococcal disease is unusual; however, infection has been reported (Boutet et al., 2001; Sejvar et al., 2005; Miller, 2012). Good laboratory practice reduces the risk of transmission for laboratory workers (Sheets et al., 2014). Thus, training and correct handling of cultures in safety cabinets is fundamental because aerosol formation of isolates when working on an open laboratory bench could be a source of infection (Sejvar et al., 2005; Borrow et al., 2014).

In addition, laboratory personnel must use appropriate personal protective equipment and meningococcal vaccination is recommended for staff that work with *Neisseria meningitides* (Sheets et al., 2014).

### Parasites

Parasitic infections have decreased in recent years. However, they have arisen due to the globalization of trade and travel (Jenkins et al., 2012). Because of the increased interest in parasitic diseases, research in both human and veterinary medicine, mainly in the context of One Health (Oura, 2014), the potential exposure to parasites in the laboratory probably increases the risk for acquiring parasitic infections. Parasitic LAIs by malaria, leishmaniasis, trypanosomiasis, toxoplasmosis, fascioliasis, or schistosomiasis among others have been reported (Herwaldt and Juranek, 1993; Herwaldt, 2001; Kinoshita-Yanaga et al., 2009; Felinto de Brito et al., 2012). The common route of parasitic LAIs has mainly been associated with needlestick injuries, although other causes such as barehanded work or research in the open field have also been reported (Herwaldt, 2001). Training and education of the laboratory staff is necessary to prevent laboratory accidents. Since some parasitic diseases are characterized by a long asymptomatic period, laboratory staff that work with parasites should be tested periodically (Herwaldt, 2001). In addition, special cautions must be taken for women of childbearing age due to the congenital transmission characteristics of some protozoan parasites (Lopes et al., 2012).

### Virus

Today, virus research is associated with wide applications in biotechnology, such as viral diseases, new vaccines, or GMOs. The potential risk with virus has been previously described regarding vaccine production, GMOs, or xenotransplantation. Research about LAI via virus is scarce, although infections with human immunodeficiency haemorrhagic virus, West Nile Virus, Dengue, or Marburg virus, among others, have been reported (Barry et al., 1995; Gaidamovich et al., 2000; Center for Disease Control and Prevention (CDC), 2002; Britton et al., 2011; Pavlin, 2014; Wei et al., 2014). Thus, appropriate biosafety and biosecurity measures, immune control strategies, training and education, and specific laboratory facilities are necessary to decrease the potential risk of LAIs and/or viral outbreaks (Lipsitch and Bloom, 2012).

## Lessons Learned to Apply in the Future

At regional and international levels, a number of countries have developed and adopted biosafety guidelines for biotechnology and bioengineering laboratories to guarantee the safety and decrease the risk of an outbreak. Although, several biosecurity guidelines and good laboratory practices manuals are available among these laboratories, their recommendations and procedures are basically the same. At international level, agencies such as WHO, FAO, OIE, national governments and other international agencies, and biosafety associations published biosafety manuals and guidelines in collaboration with expert groups to assist developing countries in the publication of their own biosafety manuals. Conferring authority to laboratories and project supervisors is essential. Since standards on biosafety and biosecurity vary according to the laboratory, institution, or even among countries (Le Duc et al., 2008; Lipsitch and Bloom, 2012), a homogenization of criteria is necessary to avoid discrepancies. Although the three international regulatory regimes, WHO, FAO, and OIE, have their own recommendations, an integrative approach on biosafety and biosecurity issues among them could improve the standardization of criteria (Sture et al., 2013).

It is necessary to control the ambient conditions, and the procedures practiced by laboratory staff in the manipulation of genetic of microorganisms should be in accordance with biosafety and biosecurity rules for personnel to avoid potential dissemination to the environment. Identification and characterization of potential hazards related with the genetic manipulation techniques or research for which the risks are still unknown is also necessary. However, responsibilities are also imputed to the researchers, since conforming to codes of conduct and awareness of the risks and hazards involved are their responsibility. Moreover, institutions are also responsible for providing the technical and human resources required to ensure compliance with all biosafety and biosecurity measures (Hackney et al., 2012).

Research that involves pathogenic organisms must be carried out in specific facilities with preventive measures and the proper training of laboratory personnel to avoid LAIs (Fonash, 2001). Specific training addressed to pathogenic organisms that are being manipulated and studied must be carried out (Narasimharao, 2009). Thus, the Certification Program for Biorisk Management Professionals proposed by the International Federation of Biosafety Associations should be an initial step to standardize the training and education of laboratory staff. In addition, a proper immune control strategy for the laboratory staff should be implemented (Rusnak et al., 2004).

To avoid occupational infections, knowledge of the application of correct microbiological procedures and techniques and the use of containment devices, facilities, and protective barriers is necessary.

Most risk group 4 pathogens, including certain arenaviruses and hendraviruses, are zoonotic agents that often cause severe or fatal disease in infected humans (Lipsitch and Bloom, 2012). Pathogens currently handled at biosafety level for risk group 3 and biosafety level for risk group 4 present a serious risk to anyone who works in a laboratory. Laboratories with biosecurity levels for risk group 3 and 4 must conform to the most rigorous safety protocols. Laboratory safety guidelines differ among laboratories, research institutions, or even by country, with a high heterogeneity in the preventive measures and training required for working on a specific pathogen, which is a real problem in a globalized world (Le Duc et al., 2008; Lipsitch and Bloom, 2012).

Laboratory-acquired infections are still a real threat in traditional microbiology and clinical laboratories. Reports about biotechnology and bioengineering laboratory accidents and infections are scarce compared to traditional microbiology and clinical laboratory accidents and infections (Kimman et al., 2008). However, if the prevalence is so high in traditional microbiology and clinical laboratories, it indicates that potential infections exist in new technological laboratories. In the near future of genetic science, the demand for new vaccines, antimicrobial drugs, or treatments for emergent and re-emergent diseases will involve new developments in biotechnology, and the risk of biological accidents will potentially increase. Consequently, specific training and education programs for laboratory personnel in addition to proper design and construction of laboratory facilities must be followed to avoid LAI and/or laboratory outbreaks (Mourya et al., 2014). The scientific community needs to heed the lessons learned from the past to anticipate future problems associated with biological risks and LAIs.

## Conflict of Interest Statement

The authors declare that the research was conducted in the absence of any commercial or financial relationships that could be construed as a potential conflict of interest.
